# Cellular Stress and Senescence Induction during *Trypanosoma cruzi* Infection

**DOI:** 10.3390/tropicalmed7070129

**Published:** 2022-07-11

**Authors:** Kamila Guimarães-Pinto, Jesuíno R. M. Ferreira, André L. A. da Costa, Alexandre Morrot, Leonardo Freire-de-Lima, Debora Decote-Ricardo, Celio Geraldo Freire-de-Lima, Alessandra A. Filardy

**Affiliations:** 1 Laboratório de Imunologia Celular, Instituto de Microbiologia Paulo de Góes, Universidade Federal do Rio de Janeiro, Rio de Janeiro 21941-902, Brazil; guimaraespkamila@micro.ufrj.br (K.G.-P.); rafaelferreira@micro.ufrj.br (J.R.M.F.); amorim@micro.ufrj.br (A.L.A.d.C.); 2 Instituto Oswaldo Cruz, FIOCRUZ, Rio de Janeiro 21045-900, Brazil; alexandre.morrot@ioc.fiocruz.br; 3 Faculdade de Medicina, Universidade Federal do Rio de Janeiro, Rio de Janeiro 21941-900, Brazil; 4 Instituto de Biofísica Carlos Chagas Filho, Universidade Federal do Rio de Janeiro, Rio de Janeiro 21941-902, Brazil; leolima@biof.ufrj.br (L.F.-d.-L.); celio@biof.ufrj.br (C.G.F.-d.-L.); 5 Instituto de Veterinária, Universidade Federal Rural do Rio de Janeiro, Seropédica 23890-000, Brazil; decotericardo@ufrrj.br

**Keywords:** *Trypanosoma cruzi*, Chagas disease, immunosenescence, immune system, oxidative stress

## Abstract

Chagas disease (CD) is a neglected tropical disease caused by *Trypanosoma cruzi* infection that, despite being discovered over a century ago, remains a public health problem, mainly in developing countries. Since *T. cruzi* can infect a wide range of mammalian host cells, parasite–host interactions may be critical to infection outcome. The intense immune stimulation that helps the control of the parasite’s replication and dissemination may also be linked with the pathogenesis and symptomatology worsening. Here, we discuss the findings that support the notion that excessive immune system stimulation driven by parasite persistence might elicit a progressive loss and collapse of immune functions. In this context, cellular stress and inflammatory responses elicited by *T. cruzi* induce fibroblast and other immune cell senescence phenotypes that may compromise the host’s capacity to control the magnitude of *T. cruzi*-induced inflammation, contributing to parasite persistence and CD progression. A better understanding of the steps involved in the induction of this chronic inflammatory status, which disables host defense capacity, providing an extra advantage to the parasite and predisposing infected hosts prematurely to immunosenescence, may provide insights to designing and developing novel therapeutic approaches to prevent and treat Chagas disease.

## 1. Introduction

Chagas disease (CD), or American trypanosomiasis, is an illness first described in 1909 by Carlos Chagas, who identified the disease’s causative pathogen, *Trypanosoma cruzi*, as well as its vector, triatomine insects [1]. Even after one century of discovery, CD is still considered one of the World Health Organization’s (WHO) “neglected tropical diseases” (NTDs) [2]. Chagas disease primarily affects low-income populations [3] and remains a social and public health problem, particularly in Latin American countries. CD is a leading cause of morbidity and mortality in tropical developing countries [4] and, due to the current unavailability of vaccines and effective treatments, vector control and alternative modes of transmission-based prevention programs are required [5].

During its complex life cycle, *T. cruzi* can switch between invertebrate hosts, which work as vectors for transmission, and vertebrate hosts, where infection takes place in a wide range of non-immune and immune cells through its obligate intracellular replication [6].

## 2. Parasite-Host Interaction in Chagas Disease

*Trypanosoma cruzi* infection is initiated when the triatomine insect vector deposits infective metacyclic trypomastigotes with its feces or urine on the host’s skin and infects cells from the mammalian host’s epithelial/mucosal barriers after a blood meal [6]. Although this early interaction between host cells and parasites may determine the outcome of *T. cruzi* infection, considerable attention and most of the studies are focused on macrophage infection, which is responsible for triggering immune responses [7].

To survive and establish a productive infection in macrophages, phagolysosome-restricted intracellular parasites must overcome the intense oxidative burst induced by the activation of macrophage membrane-associated NADPH oxidase and SLAMF1 [8], which together are responsible for the production of both reactive oxygen species (ROS) and reactive nitrogen species (RNS). The macrophage phagocytosis of trypomastigotes elicits superoxide radical (O_2_^−^) production, which is converted into H_2_O_2_ by superoxide dismutase (SOD) within the phagosome [9]. H_2_O_2_ itself, or its conversion into hydroxyl radicals (OH), has toxic effects on parasites, due to its broad reduction power [9]. Despite the powerful oxidant properties of ROS, *T. cruzi* resists an oxidative burst-dependent killing by expressing an arsenal of antioxidant enzymes holding oxidative/nitrative species detoxification capacity, through the delivery of reducing equivalents generated from NADPH via dithiol trypanothione and the thioredoxin homolog tryparedoxin [10,11,12], allowing the parasite to escape to the cytoplasm and differentiate into amastigote forms. After several rounds of amastigote duplication, the rupture of the host cell membrane and the release of infective trypomastigotes allow them to infect neighboring cells and reach the bloodstream [13]. Once in the bloodstream, parasites’ recognition and elimination by the complement system is again undermined by their expression of numerous proteins, such as calreticulin and GP160 [14,15], which hamper trypomastigotes’ complement-mediated lysis, bypassing this immune system barrier [16].

Furthermore, trypomastigotes trigger innate immune responses by the recognition of several pathogen-associated molecular patterns (PAMPs), expressed on their surface by toll-like receptors (TLRs) expressed in the host cells. This signaling pathway orchestrates an immune defense by nuclear factor kappa B (NF-kB) activation, which triggers high levels of cytokine and chemokine production, in macrophages, natural killer (NK) cells, and dendritic cells (DCs) [17]. The production of IL-12 and interferon-gamma (IFN-γ) by DCs and NK cells, respectively, elicits the crosstalk between the innate and acquired immunity required for T helper 1 (Th1) and plasma B cell clonal expansion and differentiation [18], as well as activation of parasite-specific cytotoxic CD8^+^ T cells, licensed to destroy infected cells [19]. IFN-γ derived from Th1 or CD8^+^ T, along with tumor necrosis factor α (TNF-α) and IL-12 derived from innate immune cells, activates the expression of the inducible nitric oxide synthase (Nos2) and the production of higher levels of nitric oxide (NO) by macrophages, which promotes intracellular parasite killing [20]. Recruited myeloid-derived suppressor cells (MDSCs) also play an important role in parasite replication control by upregulating Nos 2 and NO [21].

In addition, ROS production is an important factor in parasites’ replication control [22,23]. The most prominent oxidative molecule gives rise to the reaction of macrophage-derived O_2_^−^ with NO, the peroxynitrite [9]. Non-immune cells, such as cardiomyocytes [24,25], epithelial [6], and endothelial [26], are also infected and respond to *T. cruzi* infection by producing proinflammatory cytokines and undergoing an oxidative burst to control parasite replication. Although one of the main characteristics of *T. cruzi* infection is the induction of an intense Th1-inflammatory response to eliminate the parasite and promote host protection, this response is also linked with excessive immune system stimulation and disease progression, mainly during the chronic phase [18,27]. A delicate balance mediated by Th2 responses should occur to control the magnitude of the inflammatory response during *T. cruzi* infection by reducing macrophage and DC activation [28].

## 3. Oxidative Stress Response-Induced Senescence

Stress activators include many immune and pathogen-derived molecules. The intensity of stress exposure might result in distinct cell fates. In general, low-stress intensity exposure results in effective damage repair and cell cycle resumption, whereas high exposures might trigger cell apoptosis. Cells are frequently unable to repair all of the damage induced by severe and persistent sub-cytotoxic stress exposures, resulting in premature cell cycle arrest and senescence [29,30]. The term “cellular senescence” was coined by Hayflick and colleagues to characterize the progressive loss of proliferative potential of viable cells in culture after several rounds of cell division, regardless of the nutrient availability of the culture medium [31]. Senescence is mainly induced by the cellular stress response, although some terminally differentiated cells may lose their proliferative capacity as a result of their developmental program to become effector cells [32].

Over the years, besides proliferative exhaustion, new insights about how senescence could be triggered have emerged [33,34]. The continuous activation of DNA damage response (DDR) signaling caused by mitochondrial dysfunction and sustained ROS exposure are thought to be important senescence induction factors [35]. Cell DNA is the primary molecule affected by oxidative molecules; and continuous exposure to oxidant molecules may be harmful to the host cells due to their tissue- and DNA-damaging properties, which can be cumulative and irreversible [36]. When reactive species production exceeds the host’s antioxidant defense mechanisms, oxidative-induced DNA modifications occur [36]. One of these is activating the DDR, which deals with cytotoxic oxidant damage and ensures cell genetic stability through cell cycle progression arrest, as well as mechanisms to promote and coordinate DNA repair machinery [37]. Once triggered, DDR activates the damage sensor proteins ATM (ataxia telangiectasia mutated) and ATR (ataxia telangiectasia and Rad3-related) kinases, which respond to DNA double-strand breaks (DSB) and single-stranded DNA (ssDNA) [38,39], preventing cell S-phase entry via activation of p53 and upregulation of cell cycle arrest effectors, such as p21 [40]. The activation of these pathways may promote either DNA repair, senescence, or apoptosis of the infected cells [6].

Even when damaged and with their cell cycle arrested, senescent cells remain metabolically active [41]. They function as a source of ROS, which not only reinforces the senescent phenotype but also increases ROS production, triggering DNA damage and senescence in neighboring cells [42,43]. In addition to ROS, the expression of several proteins, such as cytokines, chemokines, growth factors, and interleukins is another hallmark of senescence [44]. This secretome alteration, called senescence-associated secretory phenotype (SASP), enables senescent cells to influence their microenvironment and to communicate with neighboring cells [45].

The production of high levels of ROS and senescence are linked to aging [46]. Growing evidence suggests that aging mechanisms can arise from different processes, such as cellular senescence and their SASP, neuro-immune-endocrine alterations, and accumulation of damage, although these pathways may often crosstalk [47]. Aging also compromises the immune system by reducing its effectiveness, a process called immunosenescence, which causes mild hyperactivity of innate immunity and the decline in adaptive immunity [48,49]. However, individuals carrying chronic infections can also develop premature immunosenescence, independent of chronologic age [50].

Although SASP contributes to the maintenance of the senescent state and avoids senescent cell accumulation via immunosurveillance—clearance by the innate immune system—these same factors may also have detrimental properties that, under certain conditions, might induce tissue dysfunction and tumorigenesis [44]. In contrast to apoptosis, where injured cells are rapidly phagocytosed and eliminated without triggering inflammation [51], senescent cell-derived SASP may foster local and systemic sterile inflammation, as well as the progression of chronic diseases, especially if the immune system’s ability to remove or prevent senescent cell development is overwhelmed [52].

## 4. Immunosenescence Induced by *T. cruzi* Infection-Mediated Stress and Inflammatory Responses

Regardless of the role of host cell-produced reactive species in *T. cruzi* killing, studies have shown that the parasite subverts the oxidative stress in its favor to maintain its own survival within the host cytosol [53,54]. Particularly, data from our group suggest that *T. cruzi* also explores infection-derived oxidative stress as an additional advantage to prevent its elimination and to establish its intracellular niche that contributes to the disease progression. Particularly, we found that cellular stress elicited by *T. cruzi* infection in the early stages of infection inhibits fibroblast proliferation and induces a senescent phenotype [55]. Furthermore, experimental evidence demonstrated that cells under senescence are apoptosis-resistant [56,57], and our findings suggest that senescent fibroblasts act as a long-term reservoir of parasites, allowing *T. cruzi* to replicate and establish a chronic infection [55]. Although our group correlated for the first time the early induction of senescence in *T. cruzi* infected fibroblasts, previous studies have reported the induction of immunosenescence on T cell subsets in both chronically infected human subjects [58,59] and mouse models of *T. cruzi* infection [8].

In agreement, oxidative stress induced by *T. cruzi* infection appears to modulate the expression of genes related to stress responses and cell cycle control, either by activating DDR signaling pathways or inducing suppression of cellular proliferation via cell cycle arrest [6,60,61]. It was also reported that high levels of mitochondrial ROS, combined with IFN production, cause persistent mitochondrial disturbance, boosting both ROS production and damage in cardiomyocytes, resulting in sustained inflammatory immune responses during CD [62,63]. Furthermore, infection-induced nitro-oxidative damage causes DNA breaks in cardiomyocytes and the production of TNF and IL-1β [25,64,65]. Because of its diverse damage potential, oxidative stress is increasingly being implicated as a promoter of disease progression and the development of cardiomyopathy [23,66,67]. Data from the BENEFIT benznidazole clinical study showed that CD development cannot be prevented after the harm caused by the infection has been established, even after parasitological blood clearance [68]. Overall, long-term sustained generation of *T. cruzi*-mediated nitro-oxidative stress damages cell and mitochondrial function, highlighting oxidative damage as a crucial pathogenic component in the genesis and progression of Chagas’ heart failure [54].

Aside from oxidative stress, T cell subsets and Th1 responses play an important role in inducing and mediating protective immunity during acute and chronic *T. cruzi* infections, which improves the outcome of CD pathology [18]. Since a higher parasite load and disease exacerbation have been shown in T-cell deficient mice, infection control and *T cruzi* elimination require a complex immune response of both CD4^+^ and CD8^+^ T cells [69]. Even when these cells are properly and sufficiently activated to promote infection control, parasites remain in the host’s organism for several years, activating its immune system. Data from the literature have shown that trypanocidal therapies in human and animal studies do not completely clear *T. cruzi*, supporting the notion that remaining parasites may act as a continuous source of inflammatory stimulus [6]. Furthermore, parasites may successfully hide in the organism, a state called *T. cruzi* dormancy, which allows parasite persistence even after treatment [9].

In a similar way to what has been described in chronic viral infections [70], persistent exposure to *T. cruzi*-derived antigens has been linked to a significant and progressive loss of immune functions. These events occur primarily in the T cell subsets, as evidenced by the high frequency of terminally differentiated T cells, upregulation of inhibitory receptor coexpression, and impaired cytokine production (IL-2, TNF-α, and IFN-γ), as well as cytotoxic activity. Immune exhaustion resembled immunosenescence phenotypes and was found in CD8^+^ T cells of chronic patients with CD and cardiac symptomatology, when compared to those in the indeterminate stage of the disease [58,71,72]. The same phenomenon was observed in CD4^+^ T cells from patients with severe forms of CD, which also express CD57, a marker associated with sustained antigen stimulation, replicative senescence, and T cell immune aging [59]. This suggests that the interconnection between the stage of T-cell differentiation, the individual’s inflammatory decompensation, its genetic susceptibility, and the extent of protective host responses may be strongly associated with advanced pathology and heart disease development and severity during Chagas disease (Appendix A).

## 5. Concluding Remarks

Regardless of the beneficial role of senescence induction in tumor suppression to avoid additional mutagenic effects in the DNA by blocking tumor growth via proliferative arrest and SASP-mediated clearance of tumor cells by immune cells [73,74], uncontrolled senescence has a negative impact on the organism. Paradoxically, uncontrolled *T.cruzi*-mediated chronic immune activation and immunosenescence may eventually result in impaired immune responses. The skewed activation profile of T effector cells towards non-competent parasite-specific T cells, together with degeneration of heart tissue, may directly impact more severe forms of disease pathology and symptomatology. The breakdown in host-parasite balance, combined with dampened and exhausted immune responses, highlights the potential of investigating the role of senescence in chronic diseases, specifically Chagas disease, as an open door to the design of new therapeutic strategies to prevent and efficiently treat infected individuals.

## Figures and Tables

**Figure A1:**
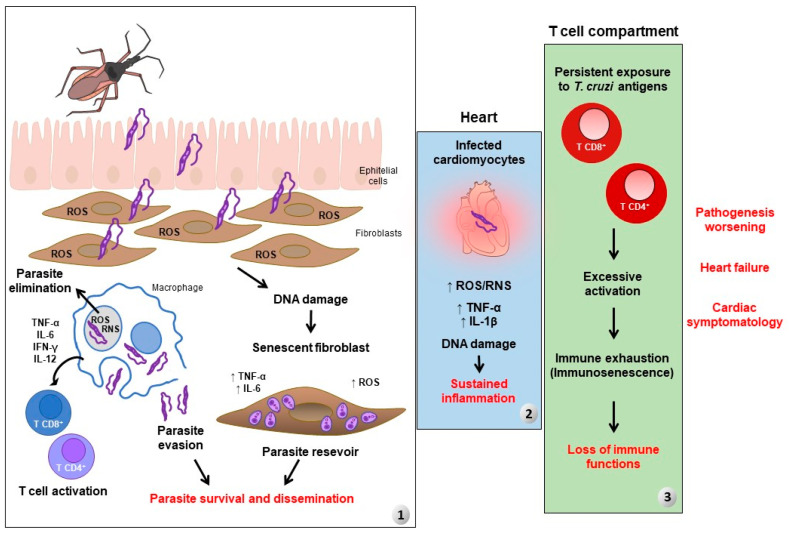
*Trypanosoma cruzi*-derived oxidative stress responses in cellular senescence induction. (**Panel 1**) *Trypanosoma cruzi* infective metacyclic trypomastigotes are released along with the feces of the triatomine insect vector during its blood meal; and they can infect cells in the skin and dermis. Infected fibroblasts produce a high level of ROS, which irreversibly damage their DNA and induce a cellular senescence phenotype, allowing them to serve as a source of inflammatory mediators (TNF-α, IL-6, and ROS) and a parasite reservoir. Infected macrophages also respond to the infection by producing ROS, RNS, and pro-inflammatory cytokines that aim to eliminate the parasite and activate adaptive immune responses against *T. cruzi*. (**Panel 2**) Infection-induced oxidative stress also damages cardiomyocyte DNA, increasing heart inflammation via the production of pro-inflammatory cytokines (TNF-α and IL-1β), which has a negative impact on heart function and disease pathogenesis. (**Panel 3**) Parasite immune evasion mechanisms allow it to survive and spread in the host’s organism, leading to sustained and excessive immune activation, promoting immune exhaustion and immunosenescence phenotypes in T CD8^+^ and T CD4^+^ cells. ROS: reactive oxygen species, RNS: reactive nitrogen species (RNS).
